# Hybrid PET/MR imaging for the prediction of left ventricular recovery after percutaneous revascularisation of coronary chronic total occlusions

**DOI:** 10.1007/s00259-020-04877-w

**Published:** 2020-05-30

**Authors:** Teresa Vitadello, Karl P. Kunze, Stephan G. Nekolla, Nicolas Langwieser, Christian Bradaric, Florian Weis, Salvatore Cassese, Massimiliano Fusaro, Alexander Hapfelmeier, Thorsten Lewalter, Markus Schwaiger, Adnan Kastrati, Karl-Ludwig Laugwitz, Christoph Rischpler, Tareq Ibrahim

**Affiliations:** 1 Klinik und Poliklinik für Innere Medizin I, Klinikum rechts der Isar, School of Medicine, Technical University of Munich, Ismaningerstr. 22, 81675 Munich, Germany; 2 Nuklearmedizinische Klinik und Poliklinik, Klinikum rechts der Isar, School of Medicine, Technical University of Munich, Munich, Germany; 3 Deutsches Herzzentrum München, School of Medicine, Technical University of Munich, Munich, Germany; 4 Institute of Medical Informatics, Statistics and Epidemiology, School of Medicine, Technical University of Munich, Munich, Germany; 5 Osypka Herzzentrum, Internistisches Klinikum München Süd, Munich, Germany; 6 DZHK (German Centre for Cardiovascular Research), partner site Munich Heart Alliance, Munich, Germany; 7 Department of Nuclear Medicine, University Hospital Essen, University of Duisburg-Essen, Essen, Germany

**Keywords:** Coronary chronic total occlusion, Viability, Hybrid imaging, PET/MR

## Abstract

**Purpose:**

To evaluate myocardial viability assessment with hybrid 2-deoxy-2-[^18^F]fluoro-d-glucose positron emission tomography/magnetic resonance imaging ([^18^F]FDG-PET/MR) in predicting left ventricular (LV) wall motion recovery after percutaneous revascularisation of coronary chronic total occlusion (CTO).

**Methods and results:**

Forty-nine patients with CTO and corresponding wall motion abnormality (WMA) underwent [^18^F]FDG-PET/MR imaging for viability assessment prior to percutaneous revascularisation. After 3–6 months, 23 patients underwent follow-up MR to evaluate wall motion recovery. In total, 124 segments were assigned to the CTO territories, while 80 segments displayed impaired wall motion. Of these, 68% (54) were concordantly viable in PET and MR; conversely, only 2 segments (2%) were assessed non-viable by both modalities. However, 30% showed a discordant viability pattern, either PET non-viable/MR viable (3 segments, 4%) or PET viable/MR non-viable (21 segments, 26%), and the latter revealed a significant wall motion improvement at follow-up (*p* = 0.033). Combined imaging by [^18^F]FDG-PET/MR showed a fair accuracy in predicting myocardial recovery after CTO revascularisation (PET/MR area under ROC curve (AUC) = 0.72, *p* = 0.002), which was superior to LGE-MR (AUC = 0.66) and [^18^F]FDG-PET (AUC = 0.58) alone.

**Conclusion:**

Hybrid PET/MR imaging prior to CTO revascularisation predicts more accurately the recovery of dysfunctional myocardium than PET or MR alone. Its complementary information may identify regions of viable myocardium with increased potential for functional recovery.

## Introduction

Percutaneous coronary intervention (PCI) of chronic total occlusion (CTO) represents one of the major challenges in interventional cardiology [1]. Revascularisation of a CTO should be considered through an individualized risk-benefit assessment encompassing clinical, angiographic as well as technical considerations [2]. Prior to CTO revascularisation, the 2018 ESC/EACTS Guidelines recommend the sought of evidence of viability, in the presence of regional wall motion abnormalities (WMA) within the CTO territory [3]. Assessment of myocardial function, viability and ischemia, by means of a reliable diagnostic test, helps in predicting the outcome of the successful revascularisation of a CTO [4], such as functional improvement. Therefore, performing this additional risk assessment in the clinical routine allows an improved patient selection for the procedure [5].

A variety of diagnostic techniques have been introduced for the assessment of myocardial viability, allowing identification of ischaemic myocardium with potentially reversible contractile dysfunction. [^18^F]FDG-PET, the clinical “gold standard” for myocardial viability, identifies viable myocardium by non-invasive visualisation of glucose metabolism. Ischaemic and dysfunctional, yet still viable myocardium presents with a preserved or even increased glucose uptake, which can benefit from revascularisation [6]. In recent years, contrast-enhanced cardiac MR imaging has been increasingly used to assess myocardial viability. Given its high spatial resolution, it allows the acquisition of both cardiac function and transmural definition of myocardial scarring, the latter using mainly late gadolinium enhancement (LGE) methods.

Fully integrated PET/MR systems allow the simultaneous acquisition of both modalities and offer the unique opportunity to merge PET and MR features both spatially and temporally, combining high-resolution anatomy and high sensitivity for the detection of molecular targets, as well as highly time-resolved functional parameters such as wall motion. In the past, both approaches produced valuable data for the prediction of functional outcome after revascularisation [7], but synergistic effects are still unknown. Moreover, in patients with CTO, studies investigating these techniques with respect to clinical outcome are scarce. Therefore, the aim of this study was to determine whether hybrid [^18^F]FDG-PET/MR allows for more accurate prediction of regional LV recovery after successful percutaneous revascularisation of a CTO in symptomatic patients in comparison with PET or MR alone.

## Methods

### Patient population

Between February 2016 and January 2018, we prospectively enrolled 49 consecutive patients with symptomatic coronary artery disease (CAD) (angina or angina equivalent) in the presence of a CTO of a relevant coronary artery (segment 1, 2, 6, 7, 11 or 13, diameter > 2.5 mm) and evidence of corresponding WMA, assessed by echocardiography or angiography. Recruitment was performed at 3 different institutions: Klinikum Rechts der Isar, Deutsches Herzzentrum and Osypka Herzzentrum in Munich. All patients underwent hybrid [^18^F]FDG-PET/MR imaging before CTO revascularisation. Exclusion criteria included contraindications for PET/MR (pregnancy, hemodynamic instability, estimated glomerular filtration < 30 ml/min, allergy to contrast agent, claustrophobia, presence of pacemakers, ICDs or any other ferromagnetic material in the body). To evaluate the impact of the procedure on the LV function, a follow-up MR was scheduled within 6 months after revascularisation and was obtained in *n* = 23 patients. Follow-up imaging was not performed in 26 patients due to different reasons including unsuccessful revascularisation (*n* = 15), not performed revascularisation (*n* = 6), implantation of MRI-incompatible defibrillator (*n* = 1) or loss to follow-up (*n* = 3). One patient had to be discarded from the analysis because of poor image quality of the PET scan (insufficient [^18^F]FDG uptake of the heart).

The study was approved by the local ethics committee, in agreement with the ethical standards according to the Declaration of Helsinki. For all patients, written informed consent was obtained.

### Imaging protocol

Imaging was performed using a hybrid PET/MR system (Biograph mMR, Siemens Healthcare GmbH, Erlangen, Germany), which acquires simultaneously PET and MR data. The MR component consists of a 3T scanner, while the PET component is built of LSO crystals equipped with avalanche photodiodes [8].

#### PET imaging

The [^18^F]FDG study was performed without the addition of a perfusion study, with a 50% relative uptake compared with remote myocardium to indicate viability, as previously described in literature [6, 9]. To optimize glucose uptake in the heart and to standardize the metabolic environment in all patients, a hyperinsulinaemic-euglycaemic clamp procedure was performed [10]. The insulin pump was prepared with 0.06 U/kg body weight/h insulin (or 0.1 U/kg body weight/h in diabetic patients) diluted in 50 ml 0.9% NaCl solution. Meanwhile, plasma glucose was determined every 10 min. After stabilisation of the plasma glucose level for approximately 60 min, 240–370 MBq [^18^F]FDG (4 MBq per kg body weight) was administered intravenously. Approximately 60 min after intravenous injection of [^18^F]FDG, a list-mode PET scan in 3D mode was started, applying electrocardiographic gating. Emission data were corrected for dead time, randoms, scatter and attenuation. Images were reconstructed using a 3D attenuation-weighted ordered subset expectation-maximisation iterative reconstruction algorithm with three iterations and 21 subsets, Gaussian smoothing at 4 mm full width at half maximum, a matrix size of 344 × 344 and a zoom of 1. A 2-point Dixon MR sequence was used in the attenuation correction of PET data, as previously described [11]. Since parts of the body may be truncated in the attenuation map because of the relatively small field of view of the MRI, the recovery of the missing attenuation map was assessed from PET emission data, using the maximum likelihood reconstruction of attenuation and activity technique (MLAA), as previously described [12].

#### MR imaging

The MR protocol consisted of functional and contrast-enhanced sequences. Firstly, fast gradient echo cine sequences were performed in order to assess left ventricular function. Thereafter, 0.2 mmol/kg gadopentetate dimeglumine (Magnograf; Marotrast GmbH, Jena, Germany) was administered intravenously, and after 10 min, inversion recovery-prepared T1-weighted gradient-echo pulse sequences with phase-sensitive reconstruction (PSIR) were acquired to allow assessment of LGE. Electrocardiographic triggering and breath holding were applied during image acquisitions.

### Image analysis

All modalities were analysed using a dedicated software package (Munich Heart), which allowed the depiction of quantitative uptake in polar maps, in which the 17-segment model established by the American Heart Association [13] was applied [14].

For PET analysis, the maximal uptake of [^18^F]FDG within the LV was set as reference and the segments subtended by the CTO were defined viable if their [^18^F]FDG uptake was at least 50% of the reference area [6, 15]. ‘In case of an [^18^F]FDG uptake below this threshold, the respective segment was defined as ‘PET non-viable’ [16].

MR analysis was performed independently by two experienced observers, who were blinded for the angiographic data. Global LV function parameters, including LV mass, end-systolic volume (ESV), end-diastolic volume (EDV) and ejection fraction (EF), were analysed by outlining of the LV contours on short-axis cine images. Similarly, the amount of LGE was manually traced on each short-axis slice by a freehand-ROI and calculated as percentage of the left ventricle [16]. Regional transmural extent of LGE was determined for each LV segment according to a 5-point score: 0 = none, 1 ≤ 25%, 2 ≤ 25–50%, 3 ≤ 50–75% and 4 > 75–100%. If the transmural extent of LGE was ≤ 50% (scores 0–2) of myocardial thickness, the segment was defined ‘MR viable’ and for any extent > 50% (scores 3, 4) as ‘MR non-viable’, respectively. Moreover, regional WMA was assessed at baseline and follow-up in each segment using a 5-point scale: 0 = normal wall motion, 1 = mild to moderate hypokinesia, 2 = severe hypokinesia, 3 = akinesia and 4 = dyskinesia. A decrease in the WMA score of at least 1 point was defined as contractility improvement.

### Statistics

The distribution of quantitative data is presented by mean ± standard deviation (SD) or median (interquartile range, IQR). Comparisons of baseline and follow-up measurements were performed by *t* tests for paired samples. The distribution of ordinal variables was compared between cohorts using the Mann-Whitney *U* test. Quantitative data is presented by absolute and relative frequencies. Intermethod agreement was calculated by means of Cohen’s Kappa.

Changes of regional wall motion and of measurements of LGE between baseline and follow-up were tested by the Wilcoxon signed rank test, due to deviations from the normal distribution. The diagnostic ability of PET and MR was assessed through receiver operating curve (ROC) analysis for clustered data to account for the assessment of multiple segments per patient [17]. PET and MR measurements served as predictor variables in a binary logistic regression model to compute a prognostic score through the linear predictor of the model (linear predictor = − 1.3487–0.6150 × [^18^F]FDG + 0.5909 × LGE). Hybrid imaging was compared with PET or MR results alone by use of this score. All statistical hypothesis testing has been performed on exploratory two-sided 5% significance levels. R 3.6.1 (The R Foundation for Statistical Computing, Vienna, Austria) has been used for computations.

## Results

### Baseline characteristics

Patient characteristics for the study group undergoing both imaging studies (*n* = 23) are summarized in Table 1*.* Mean age in this cohort of patients was 61 ± 9 years. Leading cardiovascular risk factor was hypertension, followed by dyslipidemia, smoking, CAD history in the family and diabetes. The majority (91%) of the patients showed a multivessel CAD, and about half of the patients (52%) suffered from a previous myocardial infarction. More than half of the PCI were performed in the right coronary artery (RCA, 56%), followed by left circumflex (LCx, 26%) and left anterior descending (LAD, 18%) coronary arteries.

**Table 1:** Baseline patients’ characteristics. Data are expressed as mean ± SD or as number of individuals and percentage of the total

	Baseline characteristics (*n* = 23)
Male sex	22 (96%)
Age (years)	61 ± 9
Body mass index (kg/m^2^)	29 ± 3
Diabetes	6 (26%)
Hypertension	21 (91%)
Smoking	13 (56%)
Dyslipidaemia	18 (78%)
Family history	6 (26%)
Multivessel CAD	21 (91%)
Previous myocardial infarction	12 (52%)
Coronary artery bypass	4 (17%)
Localisation	LAD 4 (18%)
	LCX 6 (26%)
	RCA 13 (56%)
Coronary dominance	Right 15 (65%)
	Left 2 (9%)
	Codominant 6 (26%)

### PET/MR imaging prior to revascularisation

Combined PET/MR imaging was successfully performed in all 23 patients who underwent revascularisation and follow-up imaging. In total, *n* = 391 segments were analysed. Accounting for individual anatomy based on coronary angiography, *n* = 124 segments were assigned to the CTO-subtended territory.

#### [^18^F]FDG-PET viability

The majority of all LV segments analysed by PET (*n* = 383/391; 98.0%) were viable, as defined by an [^18^F]FDG uptake higher than the 50% of the reference area. In all segments assigned to the CTO territory, the prevalence of viability was comparably high (*n* = 119/124, 96.0%). In CTO segments displaying a wall motion abnormality at baseline (*n* = 80), viability was slightly lower (*n* = 75/80, 93.8%).

#### MR imaging

The presence of any LGE was detectable in all patients, affecting 28.6% of the overall segments across the cohort (*n* = 112/391). However, about 90% of all segments (*n* = 352/391) were defined viable based on transmural LGE extent < 50%. The global extent of LGE across the left ventricle at baseline averaged 9.9 ± 8.7 ml (median 8 ml; IQR 3.4–11.4 ml), resulting in 6.3 ± 5.2% of the LV (median 5.1; IQR 2.4–10.4). CTO-subtended segments displayed a higher proportion of LGE (*n* = 68/124, 54.8%) and a significant drop in the proportion of MR viable segments (*n* = 96/124, 77.4%, *p* = 0.0003) as compared with the entire cohort.

Overall regional contractility analysis revealed an impaired wall motion in 168/391 (43.0%) segments baseline, whereas CTO-subtended segments were dysfunctional in 80/124 (64.5%). In those CTO segments displaying a WMA at baseline, the presence of any LGE was 70% (56/80 segments) and the number of MR viable segments further declined (*n* = 57/80, 71%).

**Fig. 1:**
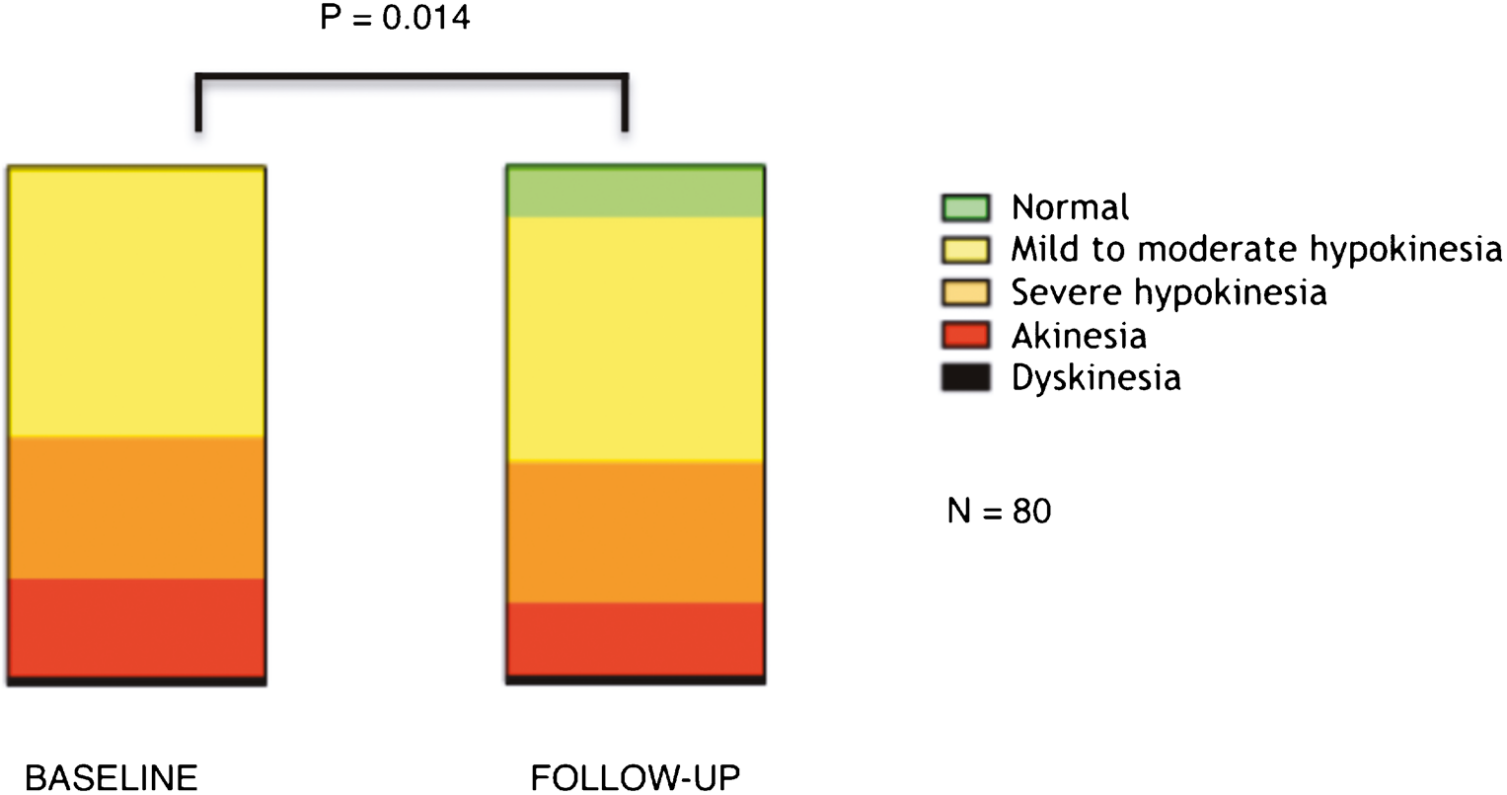
Significant improvement of WMA score between baseline and follow-up in the cohort of dysfunctional segments at baseline (*n* = 80, *p* value = 0.014)

Mean global LV EF was slightly reduced at baseline (54.6 ± 13.8%) and did not significantly change after revascularisation (58.3 ± 16.1%, *p* = 0.11). Also, volumetric parameters as well as global LGE extent showed a non-significant change after revascularisation at follow-up imaging (Table 2). After revascularisation, 28% of the initially dysfunctional segments (22/80 segments) showed significant overall improvement in regional wall motion (*p* = 0.014) (Fig. 1).

**Table 2:** Global left ventricular parameters at baseline and follow-up. All data are expressed as mean ± SD, except for LGE data, expressed as median and IQR

*n* = 23	Baseline	Follow-up	*p* value
Tissue volume	133.6 ± 34.1 ml	140.2 ± 35.5 ml	0.14
End-diastolic volume	150.2 ± 41.9 ml	146.6 ± 35.2 ml	0.57
End-systolic volume	68.9 ± 32.2 ml	63.5 ± 33.1 ml	0.22
EF	54.6 ± 13.8%	58.3 ± 16.1%	0.11
LGE extent (ml)	8 ml; IQR 3.4–11.4	7 ml; IQR 2.4–13.8	0.50
LGE extent (%LV)	5.1; IQR 2.4–10.4	4.6; IQR 2.1–9.2	0.53

#### Comparison between PET and MR imaging

PET and MR viability results for all segments are summarized in Table 3. Both modalities demonstrated concordant viability results in 89.5% of all segments resulting in a slight intermethod agreement (k .1). The overall agreement between [^18^F]FDG uptake and LGE transmurality within CTO-subtended dysfunctional segments dropped to 70% (k .04). In these segments, PET/MR concordantly identified *n* = 54 viable (68%) and *n* = 2 non-viable segments (2%). However, 30% of these segments showed a discordant viability pattern by both methods. PET viable, but MR non-viable results were detected within *n* = 21 segments (26%), and PET non-viable, but MR viable in *n* = 3 segments (4%).

**Table 3:** Distribution of the whole cohort of LV segments (above) and the subcohort of CTO-subtended dysfunctional segments (below) by the viability pattern in PET-MR

**All segments,** ***n*** **= 391**	**PET viable**	**PET non-viable**	
MR viable	347 (89%)	5 (1%)	352 (90%)
MR non-viable	36 (9%)	3 (1%)	39 (10%)
	383 (98%)	8 (2%)	
**CTO-subtended segments,** ***n*** **= 124**	**PET viable**	**PET non-viable**	
MR viable	93 (75%)	3 (2%)	96 (77%)
MR non-viable	26 (21%)	2 (2%)	28 (23%)
**CTO-subtended segments with WMA,** ***n*** **= 80**	**PET viable**	**PET non-viable**	
MR viable	54 (67%)	3 (4%)	57 (71%)
MR non-viable	21 (26%)	2 (3%)	23 (29%)
	75 (93%)	5 (7%)	

Regional wall motion differed among the different PET/MR subgroups (Table 4). PET viable/MR non-viable segments revealed a significantly more impaired WMA score at baseline than segments with concordant evidence of viability by PET and MR (*p* value = 0.019).

**Table 4:**
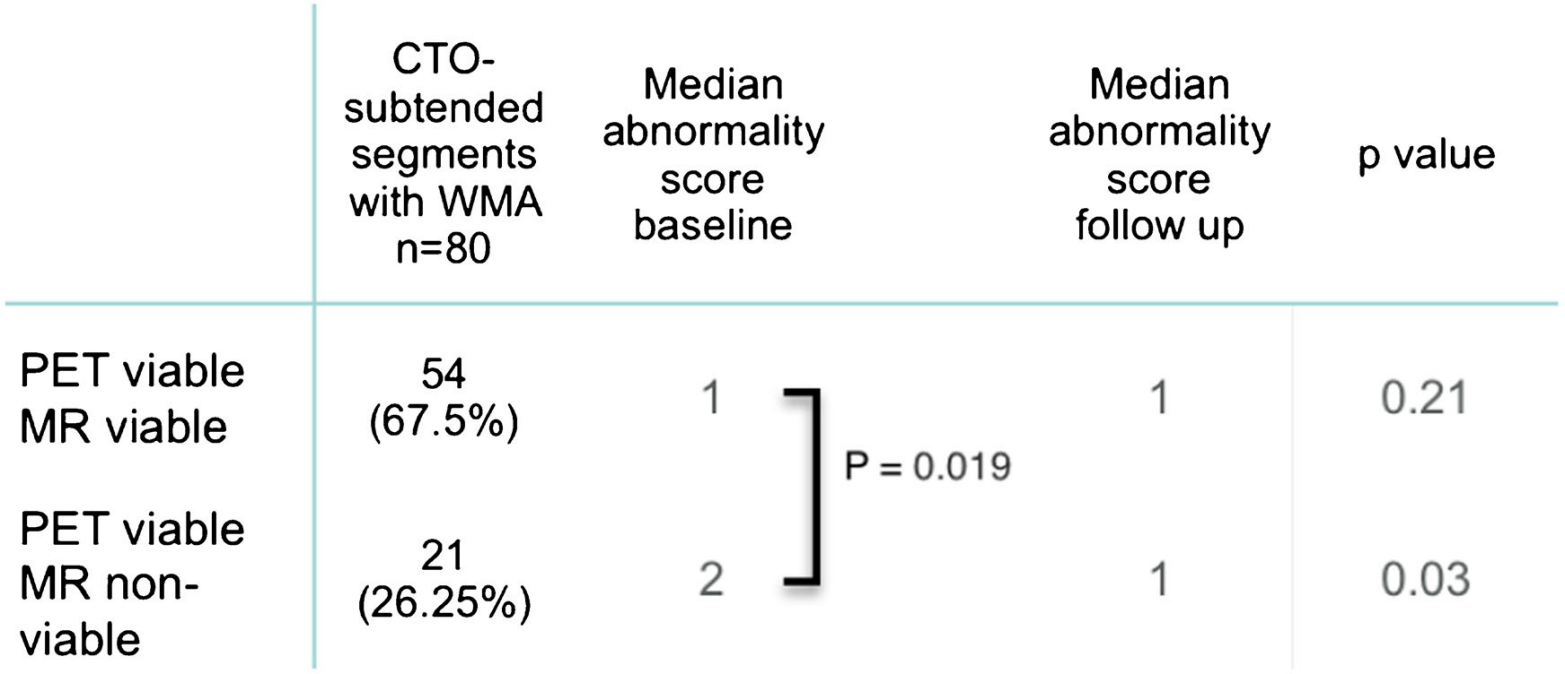
Summary of wall motion abnormality scores at baseline and follow-up of the PET viable/MR non-viable subgroup and the PET viable/MR non-viable subgroup

**Fig. 2:**
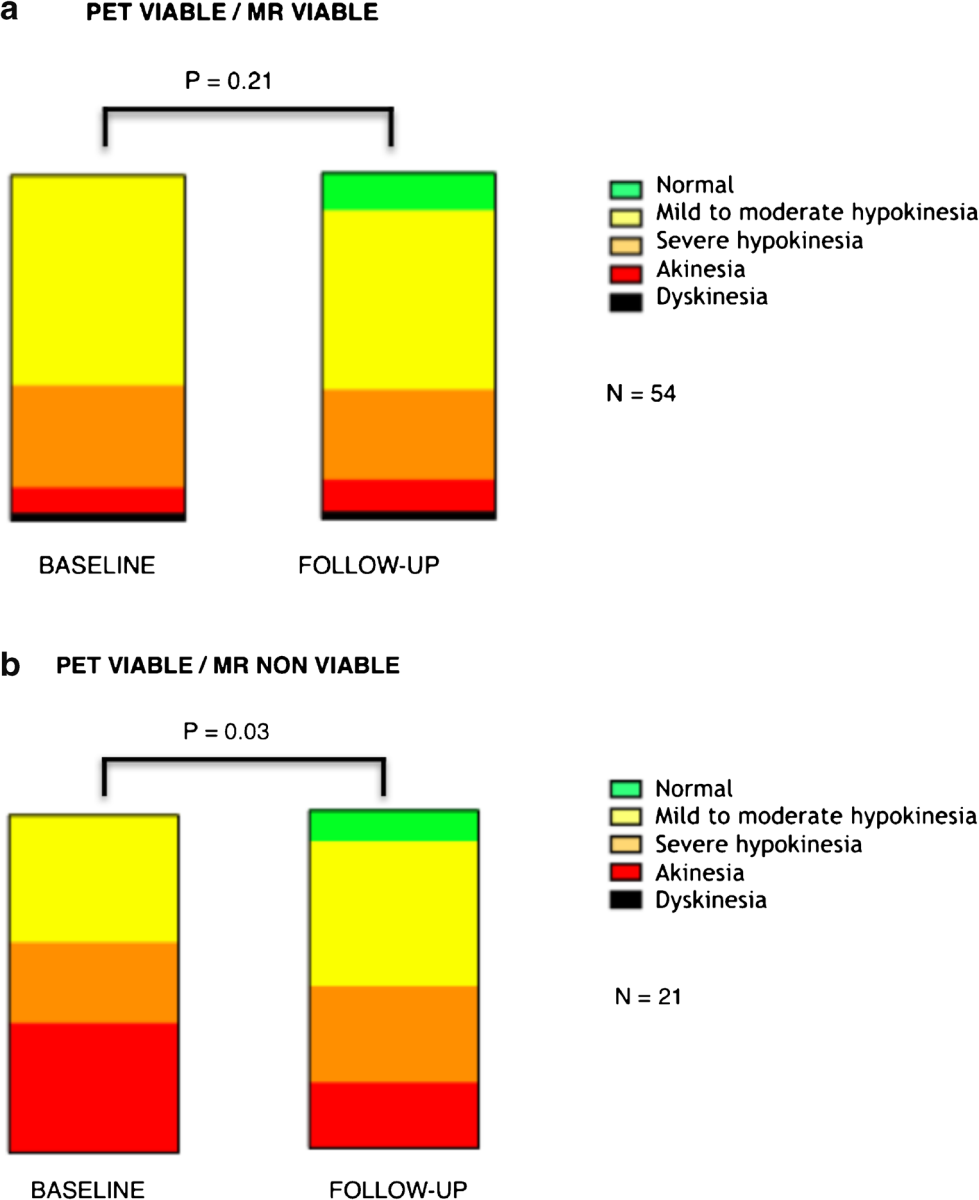
**a**, **b** WMA score at baseline and follow up in the PET viable/MR viable subgroup (**a**) and in the PET viable/MR non-viable subgroup (**b**)

While baseline to follow-up improvement of the regional WMA score within segments displaying a concordant viability by both PET and MR was not significant (*p* value = 0.21) (Fig. 2a), segments with a discordant pattern of viability (PET viable/MR non-viable) demonstrated a significant improvement of regional contractility at follow-up (*p* value = 0.033) (Fig. 2b).

#### Diagnostic value of combined PET/MR

**Fig. 3:**
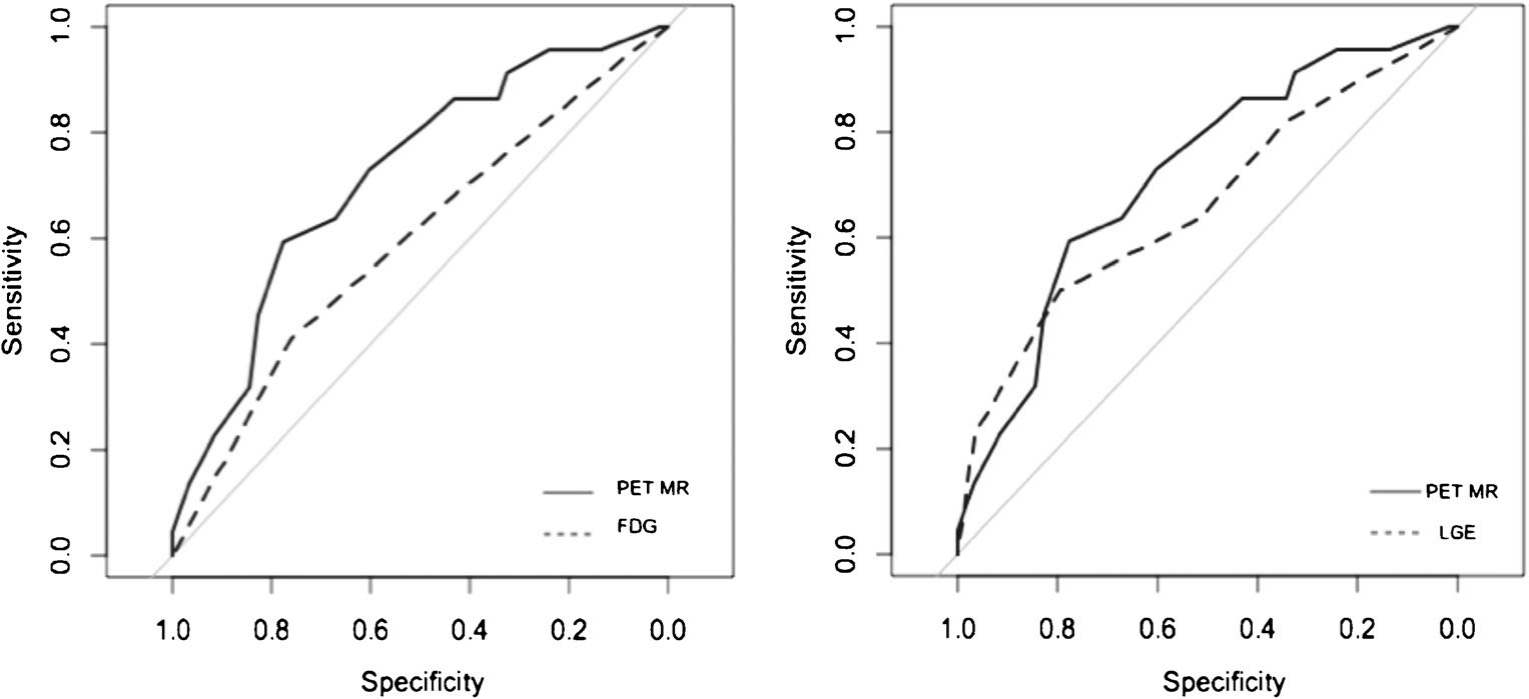
Comparison of the ROC curve obtained from the PET/MR score with PET-[^18^F]FDG (left) and LGE (right), respectively. The AUC (SE) for the PET/MR ROC curve was 0.72 (0.07), for PET-[^18^F]FDG 0.58 (0.10), and for LGE 0.66 (0.09)

Based on the ROC analysis for the prediction of wall motion recovery of the myocardial segments after CTO revascularisation, the area under the curve (AUC) for the two imaging modalities alone was 0.58 (SE 0.10) for [^18^F]FDG-PET and 0.66 (SE 0.09) for LGE-MR, respectively. The combined information of PET and MR imaging resulted in a clear improvement of the diagnostic accuracy expressed by an increase of the AUC achieving 0.72 (SE 0.07, *p* = 0.002), which was 6% superior than LGE in MR and 13% superior than [^18^F]FDG PET alone (Fig. 3).

## Discussion

**Fig. 4:**
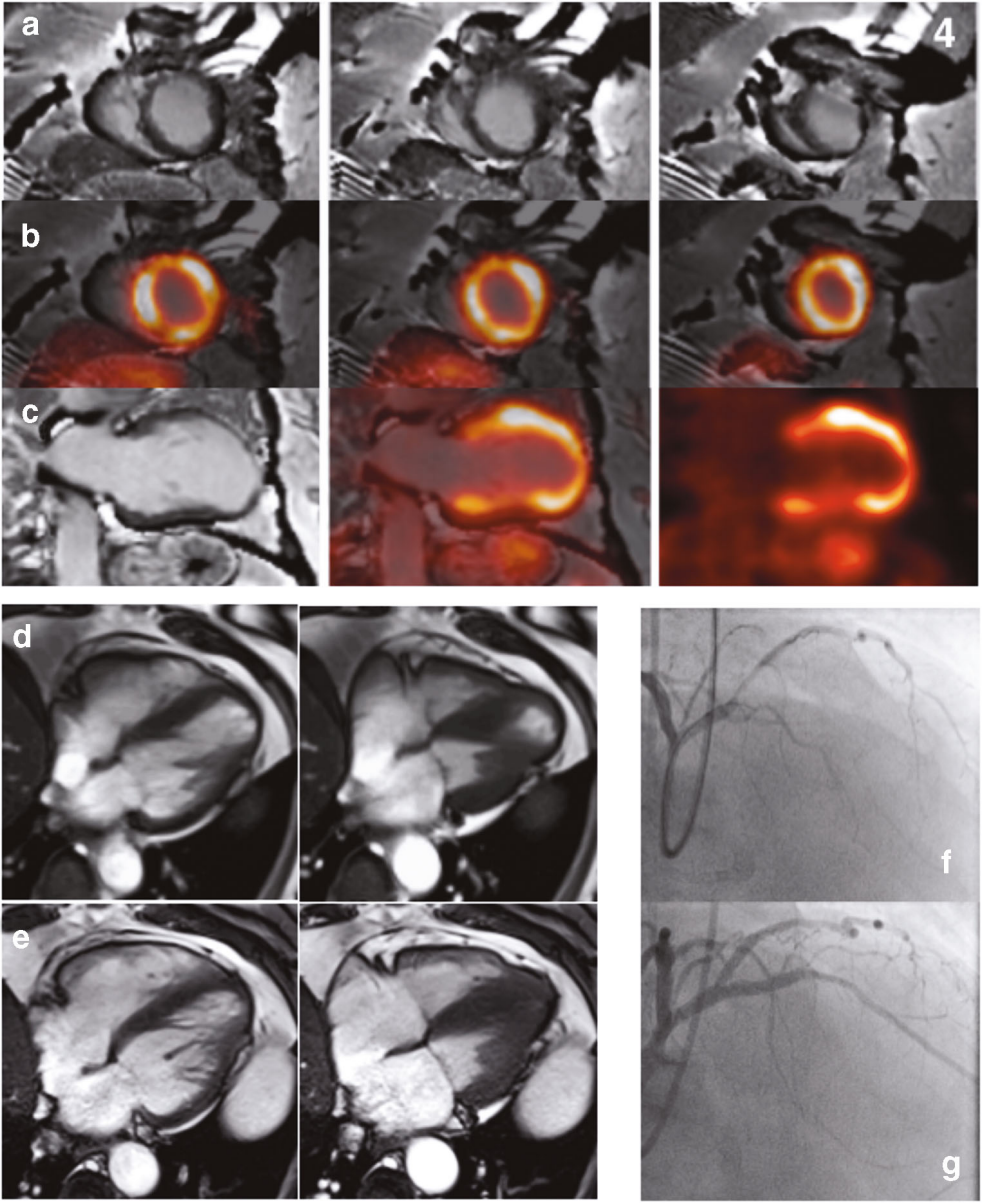
Example of a patient with a CTO of the LAD. In **a**, MR short-axis slices show a thinned anteroseptal left ventricular wall with non-transmural LGE. In **b** and **c**, PET/MR fused short axis slices showing an overall PET-viable myocardium. **c** A 2-chamber view of fused PET/MR acquisition. End-diastolic (left) and end-systolic (right) cine sequences in 4-chamber view at baseline (**d**) and at follow-up (**e**) show an improvement of the hypokinesia of the apex. Coronary angiography of the CTO of the LAD before (**f**) and after successful revascularisation (**g**)

The major finding of this study is that simultaneous hybrid PET/MR imaging predicts more accurately regional wall motion recovery after CTO revascularisation, in comparison with PET or MR alone. Combined imaging allowed the detection of dysfunctional segments with high ischaemic burden (PET viable/MR non-viable) still presenting recovery potential, which may benefit from revascularisation (Fig. 4).

Revascularisation of CTO has been associated with recovery of impaired LV function and improved survival [18, 19]. In the presence of WMA, viability imaging is recommended prior to CTO revascularisation by the 2018 ESC/EACTS Guidelines in order to determine the amount of viable myocardium and the likelihood to anticipate improvement of contractility [3]. However, registration of transmural myocardial viability based on dichotomous criteria may be challenging particularly within myocardium, since it may encompass various tissue conditions side by side, including perfused or ischaemic hibernation with or without myocardial shrinkage.

### Comparison of PET vs. MR for viability assessment

Several non-invasive imaging modalities have been introduced for the identification and assessment of myocardial viability, whereby [^18^F]FDG-PET is currently regarded as the clinical gold standard [20]. Observational evidence suggests that [^18^F]FDG-PET has the greatest sensitivity in predicting global LV functional recovery following revascularisation, compared with other imaging modalities [16]. On the other hand, techniques such as cardiac MR and stress-echocardiography may show a higher specificity [21].

In literature, regional [^18^F]FDG uptake in PET of more than 50% compared with remote myocardium and a LGE transmurality in MR of less than 50% of the ventricular wall are generally accepted markers defining viable myocardium, which have shown to have the potential to improve after revascularisation [16]. Based on these thresholds, we obtained only a slight intermethod agreement for [^18^F]FDG-PET and LGE-MR in our cohort. While 94% of the ischaemic dysfunctional CTO segments with WMA were PET viable, MR identified only 71% of these segments as viable. This discrepancy may at least partly be explained by the intrinsically higher spatial resolution of MR imaging compared with PET, allowing the detection of even small sections of transmural scar enhancement with higher sensitivity as compared with nuclear techniques, which could lead to classification as non-viable in segments with borderline findings (i.e. approximately 50% transmurality) [22]. Another fundamental difference between the two methods is that LGE MR imaging displays extracellular matrix expansion (i.e. avital tissue/oedema), while [^18^F]FDG PET maps the vital, glucose-consuming myocytes. Moreover, due to the non-specificity—from the physiological perspective—of gadolinium-based MR contrast media, the focal increase in myocardial extracellular volume as indicated by LGE can in fact be observed in various clinical pathologies including fibrosis, inflammation, oedema and cardiac storage disorders [23]. Therefore, LGE alone may present limited power in the differentiation between these tissue states, which can be increased by expansion of the MR acquisition protocol for instance by T2-weighted imaging or perfusion sequences [23]. Since we did not perform stress-MR-perfusion imaging, we were not able to reliably identify hibernating myocardium, i.e. hypoperfused, dysfunctional myocardium that is still viable and shows recovery potential after revascularisation. Adding perfusion to the viability study would increase specificity in the identification of segments with high likelihood of recovery after revascularisation, as only this approach allows to distinguish between viable and hibernating vs. viable but not hibernating myocardium [4]. Wang et al. already correlated hibernating myocardium in perfusion/metabolism PET imaging with LGE extent in patients with CTO [24]. This study showed that not only segments with non-transmural LGE had great probability of having hibernating tissue, but also one-third of segments with transmural scar in MR still showed myocardial hibernation [24].

On the contrary, [^18^F]FDG-PET definitely adds sensitivity in identifying small amounts of still viable epicardial myocardium, even in the presence of large myocardial scars (LGE > 50% of myocardial thickness). The subepicardial [^18^F]FDG activity is able to show different properties of myocardium including hibernation, stunning and normal myocardium [24].

In our cohort, we found a large prevalence, about one-quarter of CTO segments (26%), with a discordant viability pattern, mostly showing viability in PET and non-viability in MR. This combination allowed to identify those segments displaying a high regional wall motion abnormality and high potential for recovery of function after CTO revascularisation. In fact, it was the only combination (PET viable/MR non-viable) in our cohort to show a significant improvement of WMA at follow-up. Hence, this particular pattern of viability may allow the identification of highly ischaemic and dysfunctional myocardium, which could benefit substantially from revascularisation. The prevalence of this combination (PET viable/MR non-viable) was relatively high in our patient population suffering from chronic-ischaemic, mostly multivessel coronary artery disease. Conversely, in a recent study in patients with acute myocardial infarction and successful reperfusion, in which myocardial stunning may have primarily dominated, this discordant viability pattern (PET viable/MR non-viable) was not commonly described [16].

### Diagnostic value of combined PET/MR

This is the first study that sought to assess regional myocardial viability by simultaneous PET/MR imaging in patients undergoing a CTO revascularisation. Using a hybrid PET/MR scanner with integration of both modalities allows a truly synchronous/simultaneous acquisition of complementary information such as high-resolution anatomy and myocardial metabolism in merged images [25]. Thus, the principle to combine different imaging modalities in order to increase the diagnostic accuracy in predicting myocardial viability and long-term improvement of regional impaired function is highly attractive. Based on the ROC-analysis, simultaneous PET/MR imaging was superior to LGE-MR or [^18^F]FDG-PET alone in predicting functional recovery after revascularisation of CTO. Therefore, LGE-MR adds specificity to the investigation, outlining the segments with more pronounced wall motion dysfunction that could potentially benefit from revascularisation.

The superiority in combining the complementary information from PET and MR has been demonstrated already in other clinical settings, such as in the diagnosis of cardiac sarcoidosis [26] or prostate cancer [27], enhancing the value of hybrid PET/MR imaging in different fields.

Although we demonstrated a higher diagnostic accuracy in detecting regional functional improvement by simultaneous imaging of [^18^F]FDG-PET and MRI-LGE as compared with these modalities alone, we did not observe significant effects on global LV parameters. An explanation may be that the EF was only slightly impaired before revascularisation of the CTO and that the global extent of LGE across the left ventricle was only 6.3 ± 5.2%. Similarly, recent small- to moderate-sized CMR imaging studies which evaluated CTO showed conflicting results or only minor improvements of LVEF [28, 29]. Furthermore, the wall motion analysis used only assesses substantial improvements of the wall motion abnormality. Subtle improvements, however, are usually not captured because of the analysis through a rather “rough” 5 point score. Moreover, since a CTO usually develops and exists for months to years, it is also possible that the wall motion improvement achieved after revascularisation may only occur after a longer period of time (e.g. after 9 months or one year) than the actual follow-up. Larger studies are warranted to further assess whether revascularisation of CTO based on hybrid imaging may have an impact on more established clinical outcome measures such as LV function or even mortality and thus provide evidence to become a meaningful approach in planning such interventions.

### Limitations

This was a pilot study with a comprehensive protocol, and therefore, a relatively small number of patients were included, despite the involvement of three centres. Larger multicentre studies are warranted to confirm these preliminary results. The study population finally underwent hybrid PET/MRI, successful CTO revascularisation and a follow-up imaging, respectively. Moreover, the global LV function at baseline in this cohort was almost normal and thus the amount of dysfunctional segments in the CTO territory was moderate. Only patients with successful CTO revascularisation underwent follow-up imaging, so we cannot comment potential positive LV remodeling after CTO revascularisation in comparison with patients after failed PCI. Finally, in order to facilitate straightforward imaging, a MRI protocol solely assessing function and LGE but no further sequences such as perfusion imaging was applied, which could have improved the detection of myocardial hibernation.

## Conclusions

Hybrid PET/MR imaging prior to successful CTO revascularisation showed a better overall diagnostic accuracy than PET or MR alone in predicting regional improvement of wall motion abnormalities in territories affected by CTO. The complementary information derived from both modalities may help to identify small amounts of viable, dysfunctional and hibernating epicardial myocardium within large scars with potential to improve contractility after CTO revascularisation.

## Data Availability

The datasets generated during and/or analysed during the current study are available from the corresponding author on reasonable request.
